# Zn Doping Effect on the Performance of Fe-Based Catalysts for the Hydrogenation of CO_2_ to Light Hydrocarbons

**DOI:** 10.3390/molecules27031065

**Published:** 2022-02-04

**Authors:** Nikolay Dmitrievich Evdokimenko, Gennady Ivanovich Kapustin, Olga Petrovna Tkachenko, Konstantin Borisovich Kalmykov, Alexander Leonidovich Kustov

**Affiliations:** 1 Laboratory of Nanochemistry and Ecology, National University of Science and Technology “MISiS”, 4 Leninsky Prospekt, 119049 Moscow, Russia; kyst@list.ru; 2 Laboratory for the Development and Research of Polyfunctional Catalysts, N.D. Zelinsky Institute of Organic Chemistry RAS, 47 Leninsky Prospekt, 119991 Moscow, Russia; gik@server.ioc.ac.ru (G.I.K.); ot113@mail.ru (O.P.T.); 3 Department of Chemistry, M. V. Lomonosov Moscow State University, Leninskie Gory, GSP-1, 119991 Moscow, Russia; sirius.91@mail.ru

**Keywords:** CO_2_ hydrogenation, hydrocarbons, light hydrocarbons, heterogeneous catalyst, zinc addition, Fe-containing catalysts

## Abstract

In this work, we studied the role of zinc in the composition of supported iron-containing catalysts for the hydrogenation of CO_2_. Various variants of incipient wetness impregnation of the support were tested to obtain catalyst samples. The best results are shown for samples synthesized by co-impregnation of the support with a common solution of iron and zinc precursors at the same molar ratio of iron and zinc. Catalyst samples were analyzed by various methods: Raman, DRIFT-CO, TPR-H_2_, XPS, and UV/Vis. The introduction of zinc leads to the formation of a mixed ZnFe_2_O_4_ phase. In this case, the activation of the catalyst proceeds through the stage of formation of the metastable wustite phase FeO. The formation of this wustite phase promotes the formation of metallic iron in the composition of the catalyst under the reaction conditions. It is believed that the presence of metallic iron is a necessary step in the formation of iron carbides—that is, active centers for the formation and growth of chain in the hydrocarbons. This leads to an increase in the activity and selectivity of the formation of hydrocarbons in the process of CO_2_ hydrogenation.

## 1. Introduction

Every year, the issue of reducing greenhouse gas emissions is becoming more acute. One such gas is CO_2_. The concentration of carbon dioxide is constantly growing every year and, in 2019, reached a record value for the last 20 million years [1,2]. In recent years, an increasing number of researchers have turned their attention to developing ways to reduce carbon dioxide emissions. Of great interest is the chemical conversion of CO_2_ into valuable products that could subsequently be reused in various areas of the chemical industry [3,4,5]. The CO_2_ molecule is extremely stable; therefore, for carrying out chemical reactions, it is necessary to use a heterogeneous catalyst and high-energy reagents, for example, hydrogen [6]. From this point of view, hydrogenation of CO_2_ on heterogeneous catalysts is a simple and convenient way to obtain synthesis gas, hydrocarbons of various structures, methanol, other alcohols, and some oxygenates [7,8,9,10,11,12]. Usually, the conversion of CO_2_ into value-added products involves two stages: the conversion of CO_2_ to CO by a reverse water shift reaction and the further conversion of CO by the Fischer–Tropsch process [13,14,15]. Iron-based catalysts for the Fischer–Tropsch process promote both the reverse water shift reaction and the Fischer–Tropsch process [13,14,16]. Therefore, it seems interesting to carry out this process in one stage on iron-containing catalysts. One of the key factors affecting the properties of iron-containing catalysts in the hydrogenation of CO_2_ is the ratio of the oxide and carbide phases of iron. Iron oxides are responsible for the reverse water shift reaction [17,18], and iron carbides are the centers of chain formation and growth [19,20]. Various promoters are used to increase the activity and selectivity of these catalysts; as a rule, these are alkali metals. They promote the formation of iron carbides under the reaction conditions and an increase in the selectivity of the formation of light hydrocarbons, but at the same time, they prevent the adsorption of hydrogen, thereby slowing down the course of the reaction [21,22,23]. At the same time, the introduction of zinc into iron-containing catalysts increases the selectivity of the formation of light hydrocarbons. Zinc helps to increase the activity of these catalysts in the Fischer–Tropsch process [24,25], increases the adsorption of carbon dioxide [24,26,27] and hydrogen [27,28], and increases the activity in the reverse water shift reaction [29,30]. It has been shown that zinc increases the dispersity of iron particles and acts as a structural promoter [31,32,33,34,35,36]. Recently, it has been suggested that the addition of zinc is not only a structural promoter, but also increases the stability of the iron-containing catalyst owing to electron density transfer from zinc to iron, promotes the formation of iron carbides, and suppresses the formation of magnetite Fe_3_O_4_ during the operation of the iron–zinc coprecipitated catalyst [21]. It should be noted that zinc is one of the main components of copper-containing catalysts for the hydrogenation of CO_2_ to methanol, where zinc plays the role of both a structural and electronic promoter, providing high dispersion of copper and increasing the adsorption of CO_2_ [37,38,39,40,41]. Although the ability of zinc to improve the properties of Fischer–Tropsch catalysts [24] and hydrogenation of CO_2_ to methanol has been studied in sufficient detail, the effect of zinc on the hydrogenation of CO_2_ to hydrocarbons has not been sufficiently studied. A deeper understanding of the effect of zinc on the properties of iron-containing catalysts is needed in order to design effective catalysts for the hydrogenation of CO_2_ into hydrocarbons. Therefore, the aim of this work was to study the role of zinc in the composition of iron-containing catalysts for CO_2_ hydrogenation. In this article, we report the presence of an electronic effect due to the presence of zinc in the structure of an iron–zinc catalyst deposited on a carrier ZrO_2_.

## 2. Results

### 2.1. CO_2_ Hydrogenation

An important issue for determining the role of zinc in the composition of the iron-containing catalyst for the hydrogenation of CO_2_ is the understanding of the method of introducing zinc into the catalyst structure. Zinc was introduced together with iron at the stage of co-impregnation of the support with metal precursors or successively. The results of catalytic tests of these samples are presented in Table 1. For comparison, the data on the catalytic properties of monometallic supported catalysts based on zinc and iron synthesized in a similar way on the same ZrO_2_ support are presented. The 5%Zn/ZrO_2_ sample demonstrates extremely low activity in CO_2_ hydrogenation, which indicates the inertness of zinc in this reaction. Most likely, this is caused by the filling of zinc d-orbitals with electrons, which are necessary for the adsorption and activation of hydrogen. Sample 5%Fe/ZrO_2_, without zinc addition, exhibits satisfactory activity, comparable to similar supported iron-containing catalysts for CO_2_ hydrogenation [42,43]. With the sequential deposition of the catalyst components, the obtained samples show a lower activity than the sample containing only iron. Probably, in the case of sequential deposition of iron at the first stage and zinc at the second stage, the iron turns out to be isolated by zinc from the reaction region in the surface layers of the catalyst and is inaccessible to gaseous reagents. In the case of zinc deposition at the first stage and iron at the second, iron is isolated from the ZrO_2_ carrier, which is not absolutely inert and takes part in the activation of the CO_2_ molecule [10]. Higher activity can be achieved only in the case of obtaining a catalyst by the method of co-impregnation of the support in terms of moisture capacity with a common solution of iron and zinc precursors. In this case, iron and zinc are evenly distributed over the surface of the carrier, which ensures the efficient operation of all components of the catalyst. The main reaction products under these conditions of CO_2_ hydrogenation were carbon monoxide and water. The formation of a small amount of light hydrocarbons, mainly methane, was observed, and in the case of the sample obtained by the co-impregnation method, the selectivity of the formation of C_1_–C_10_ hydrocarbons was at a maximum compared with the rest of the samples and amounted to 9%.

Table 2 shows the results of experiments to determine the effect of the zinc content on the catalytic properties of the iron-containing catalyst for the hydrogenation of CO_2_. The rate of CO_2_ hydrogenation is higher in all samples with zinc addition than without it. However, the maximum activity is shown by samples with a ratio of iron to zinc of approximately one to one. The dependence of the selectivity of the formation of hydrocarbons on the zinc content also has an extreme character with a maximum in the region of the iron to zinc ratio of about 1:1. Thus, even at this stage, it is clear that the addition of zinc contributes to an increase in the activity of the iron-containing catalyst and the selectivity for the formation of hydrocarbons in the hydrogenation of CO_2_.

**Figure 1:**
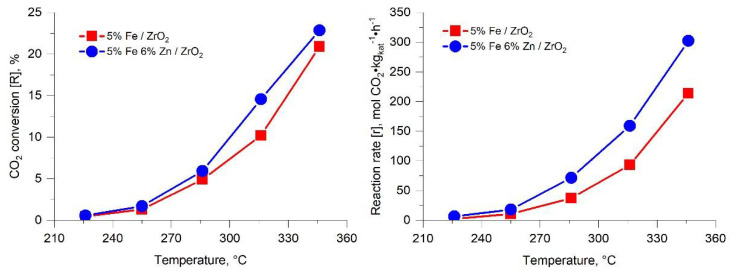
CO_2_ conversion and CO_2_ hydrogenation on iron-containing catalysts with and without zinc addition (H_2_/CO_2_ = 2:1, P = 50 atm, 600 h^−1^).

**Figure 2:**
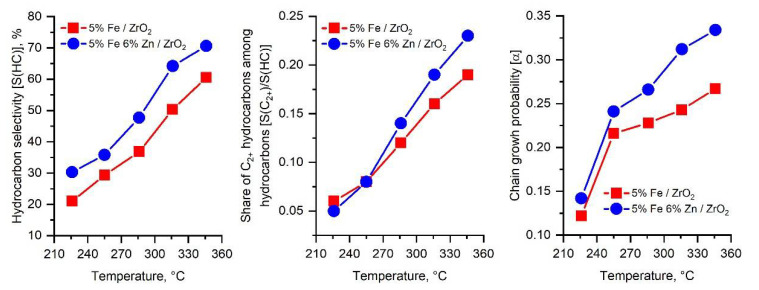
Selectivity to hydrocarbons, share of C_2+_ hydrocarbons and chain growth probability in CO_2_ hydrogenation on iron-containing catalysts with and without a zinc additive (H_2_/CO_2_ = 2:1, P = 50 atm, 600 h^−1^).

For a more detailed understanding of the effect of zinc on the properties of iron-containing catalysts in the hydrogenation of CO_2_, a sample of the composition 5%Fe 6%Zn/ZrO_2_ was chosen, obtained by impregnating the support in terms of moisture capacity with a combined aqueous solution of iron and zinc precursors. The sample 5%Fe/ZrO_2_ obtained by a similar method was used as a reference sample. The results of catalytic studies are shown in Figure 1 and Figure 2. In both samples, the hydrogenation rate increases with temperature. Among the products, the formation of carbon monoxide, water, and light hydrocarbons C_1_–C_10_ of various structures was observed. The formation of aromatic hydrocarbons, alcohols, and other oxygenates during the reaction was not observed even on an iron–zinc catalyst. Selectivity of formation of hydrocarbons C_1_–C_10_ increases with the increasing temperature. The distribution of hydrocarbons along the length of the carbon chain in all cases obeys the Schultz–Flory equation. The sample with the addition of zinc exhibits high activity in the hydrogenation of CO_2_ and selectivity for the formation of light hydrocarbons C_1_–C_10_. In this case, the probability of chain growth on an iron–zinc sample turns out to be 15–25% higher than on a catalyst sample that does not contain zinc. The data obtained unambiguously indicate the possibility of a significant improvement in the properties of iron-containing catalysts by introducing zinc into their structure.

### 2.2. Raman Spectroscopy

**Figure 3:**
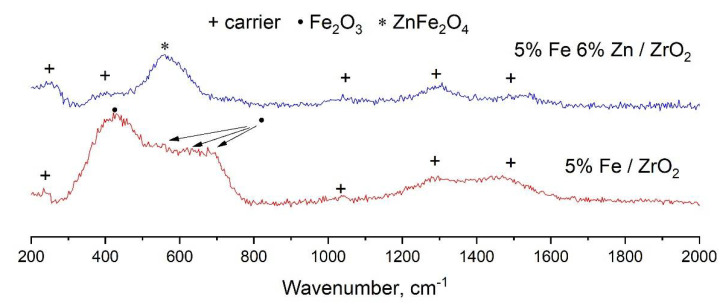
Raman spectra of iron-containing catalysts for CO_2_ hydrogenation with and without zinc addition.

The Raman spectra of 5%Fe/ZrO_2_ and 5%Fe6%Zn/ZrO_2_ catalyst samples are shown in Figure 3. Lines 260 cm^−1^, 400 cm^−1^, 1030 cm^−1^, 1280 cm^−1^, and 1528 cm^−1^ belong to the carrier components, zirconium and lanthanum oxides [44]. The spectrum of the 5%Fe/ZrO_2_ catalyst sample contains a line at 235 cm^−1^ and a wide shoulder after 510–780 cm^−1^, which corresponds to a superposition of one of the main lines of zirconium and several low-intensity lines characteristic of hematite [45]. The sample 5%Fe6%Zn/ZrO_2_ contains an intense line at about 560 cm^−1^, which clearly indicates the formation of ZnFe_2_O_4_ in the structure of this sample [44]. Raman spectra of spent catalysts after CO_2_ hydrogenation are presented in Supplementary Materials, Figure S2. Magnetite Fe_3_O_4_ lines are present in the spectrum of spent 5%Fe/ZrO_2_. It is not possible to draw conclusions from the spectrum of 5%Fe6%Zn/ZrO_2_ owing to the low intensity of the lines.

### 2.3. DRIFT-CO

**Figure 4:**
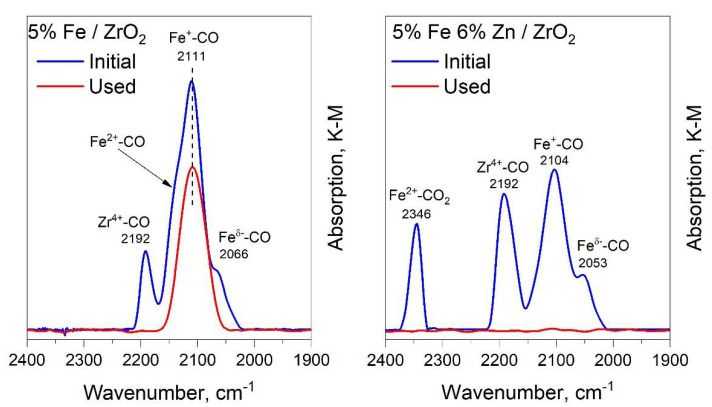
IR diffuse reflectance spectra of iron-containing catalysts with and without zinc addition.

Figure 4 shows the diffuse reflectance IR spectra of samples of iron-containing catalysts with and without zinc addition before activation and after catalytic studies. The band with a maximum at 2192 cm^−1^ belongs to the linear zirconium carbonyl Zr^4+^-CO. On the side of shorter wavelengths, under this band, there may be a band belonging to the carbonyl La^3+^-CO (about 2170 cm^−1^). In the spectrum of the sample containing zinc, a broad band with a maximum at 2192 cm^−1^ can be attributed to the super-position of two bands from the linear carbonyls Zr^4+^-CO and Zn^2+^-CO. The band at 2140 cm^−1^ in the spectra of the 5%Fe/ZrO_2_ sample belongs to the linear carbonyl on ferrous cations Fe^2+^-CO. The band at 2104–2111 cm^−1^ belongs to linear carbonyl on monovalent iron cations Fe^+^-CO. Bands at 2066–2053 cm^−1^ can characterize linear iron carbonyls Fe^δ+^-CO. Based on the fact that iron in the Fe^3+^ state does not form carbonyls, the presence of lines observed in the spectrum can be explained by the presence in the samples of a small amount of iron in lower oxidation states, which is formed during evacuation or is initially present in the catalyst structure in the form of coordination unsaturated atoms on the faces and tops of crystallites of iron compounds [45]. The band at 2346 cm^−1^ in the spectrum of 5%Fe6%Zn/ZrO_2_ refers to the carbonate Fe^2+^-CO_2_, which is formed upon the reduction of Fe^3+^ with a CO molecule. Their presence indicates a high oxygen mobility on the surface of iron crystallites and the possibility of fairly easy reduction of Fe^3+^ to a state with lower oxidation states. The 5%Fe6%Zn/ZrO_2_ sample, after reduction and catalysis, completely loses its ability to adsorb CO, while on the 5%Fe/ZrO_2_ sample, it significantly decreases. After catalysis, in the spectrum of the 5%Fe/ZrO_2_ sample, one band remains, which characterizes the Fe^+^-CO carbonyl. This can be due to the blocking of the catalyst surface by carbon reaction products. These results show that the introduction of zinc provides mobile oxygen in the structure of iron crystallites, which can lead to a deeper reduction of iron and contribute to an increase in the amount of adsorbed CO_2_ molecules under the reaction conditions.

### 2.4. TPR-H_2_

**Figure 5:**
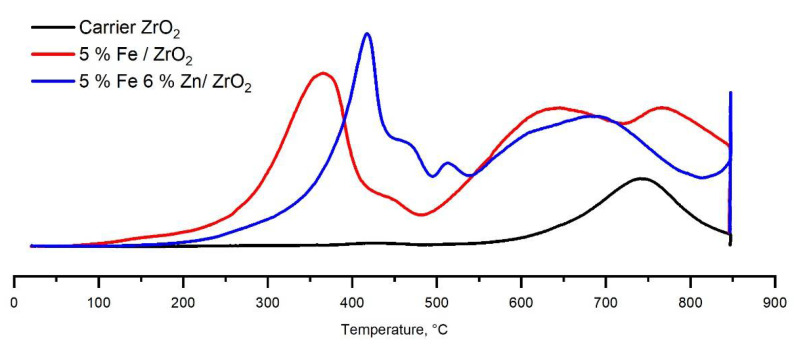
TPR-H_2_ curves of the ZrO_2_ support and samples of iron-containing catalysts with and without zinc addition.

Figure 5 shows the TPR-H_2_ curves of the ZrO_2_ support and samples of iron-based catalysts with and without zinc addition. The TPR-H_2_ curve of the catalyst support shows two peaks with maxima at temperatures of 428 °C and 745 °C. The first peak with a low intensity can be attributed to the partial reduction of lanthanum oxide La^3+^→La^2+^, and the second peak with a high intensity can be attributed to the partial reduction of zirconium oxide Zr^4+^→Zr^2+^ [46]. On the TPR-H_2_ curve of the 5%Fe/ZrO_2_ catalyst, four peaks of different intensities are observed. Two peaks with maxima at 448 °C and 767 °C refer to the reduction of the support components. The maxima of these peaks are shifted to a higher temperature region by about 20 °C compared with the position of these peaks on a pure support without iron. Two other intense peaks with maxima at 367 °C and 647 °C refer to the reduction of iron oxide to metallic iron according to the following scheme Fe_2_O_3_→Fe_3_O_4_→Fe^0^ [47,48,49]. On the TPR-H_2_ curve of the 5%Fe6%Zn/ZrO_2_ catalyst sample, the peaks of the support reduction are shifted to an even higher temperature region, 468 °C for La^3+^→La^2+^, and for the Zr^4+^→Zr^2+^ process, the peak maximum is at temperatures above 850 °C. The peak with a maximum at a temperature of 417 °C corresponds to the reduction of Fe_2_O_3_ to magnetite Fe_3_O_4_, while on the TPR-H_2_ curve of an unprocessed catalyst, the maximum of this peak is at about 367 °C. A broad peak with a maximum at 695 °C refers to the reduction of iron oxides to metallic iron, and the shoulder at 610 °C indicates that this process partially passes through the formation of a metastable wustite phase [50]. This can facilitate the easier reduction of iron to the metallic state Fe^0^ [51], and the formation of this iron is a prerequisite for the formation of Hegg carbides—that is, active centers for the formation and growth of a chain of hydrocarbons [19]. The peak with a maximum at about 515 °C can be attributed to the reduction of zinc oxide to the metallic state ZnO→Zn^0^. Zinc directs the process of iron reduction to the stage of FeO formation. Possibly, the stabilization of the FeO phase is achieved by the migration of Zn^2+^ ions to the structure of iron [51]. Table 3 shows the specific absorption of hydrogen by the carrier and catalyst samples in the course of TPR-H_2_ studies. In none of the cases is it possible to achieve the reduction of the entire amount of iron in the composition of the sample to the state Fe^0^, and the introduction of zinc does not lead to a noticeable change in the amount of absorbed hydrogen. Using such a sample, the introduction of zinc into the structure of these catalysts does not lead to a deeper reduction of iron, but changes the path of the iron activation process. It is also worth noting that, in the case of a catalyst with the addition of zinc, hydrogen is used not only for the reduction of iron, but also for the reduction of zinc. Taking into account the approximately equal amounts of absorbed hydrogen in the TPR-H_2_ process, it can be assumed that, in the case of a catalyst with the addition of zinc, although it is possible to achieve a deeper reduction of iron, the overall degree of iron reduction turns out to be lower.

### 2.5. XPS

**Figure 6:**
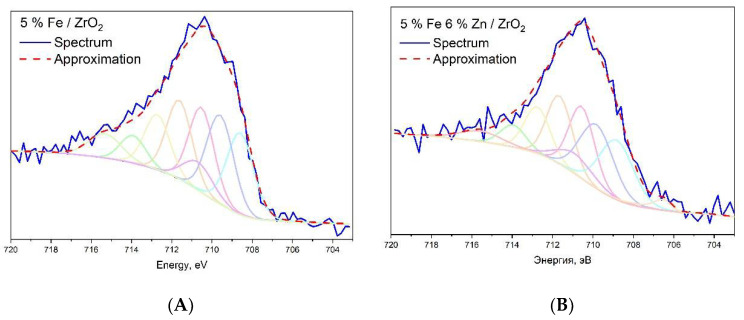
Result of approximation by Gupta–Sena multiplets of the Fe2p3/2 spectral region of catalyst samples 5%Fe/ZrO_2_ (**A**) and 5%Fe6%Zn/ZrO_2_ (**B**) after activation and hydrogenation of CO_2_ (H_2_/CO_2_ = 2:1, P = 50 atm, 600 h^−1^).

XPS spectra and spectra in the range of binding energies of iron Fe2p of samples of iron-containing catalysts with and without zinc after the reaction of hydrogenation of carbon dioxide are presented in Supplementary Materials Figure S1. The survey spectra show the presence of photoelectron lines of oxygen O1s and Auger oxygen lines O (KLL); photoelectron lines of carbon C1s, photoelectron lines of iron Fe2p, and Auger lines of iron Fe (LMM); photoelectron lines of lanthanum La3d, La4p3, and La4d; and photoelectron lines of zirconium Zr3s, Zr3p, Zr3d, Zr4s, and Zr4p. In the case of the zinc-promoted sample, the presence of photoelectron lines of zinc Zn2P1, Zn2p3, Zn3s, and Zn3p and Auger lines of Zn (LMM1), Zn (LMM2), and Zn (LMM3) is observed. The chemical composition of the surface of the catalyst samples is presented in Table 4. It can be seen that the iron content is significantly less than that calculated during the synthesis. This is because of the incorporation of iron into the surface layers of the support, which are inaccessible for observation by the XPS method. The zinc content in the sample, promoted with zinc, is lower than that calculated during the synthesis; however, in this case, the decrease in concentration can be explained by the loss of zinc as a result of the entrainment of metallic zinc during the activation of the sample and the hydrogenation of O_2_. The presence of carbon on the surface of all samples after catalysis is due to the deposition of reaction products on the catalyst surface during CO_2_ hydrogenation. The results of the approximation of the spectra of high-spin electrons of Fe2p3/2 catalysts after activation and hydrogenation of CO_2_ are shown in Figure 6 [52]. In the approximation process, the best results for all samples are achieved when using Gupta–Sena multiplets corresponding to the magnetite structure. The calculated fractions of Fe^3+^ and Fe^2+^ ions, as well as iron in the zero oxidation state Fe^0^, are presented in Table 5. It can be seen that the introduction of zinc promotes the formation of metallic iron on the catalyst surface, which is consistent with the data obtained on TPR. In the catalyst without the addition of zinc, the formation of iron in the zero oxidation state is generally not observed.

### 2.6. UV/VIS

**Figure 7:**
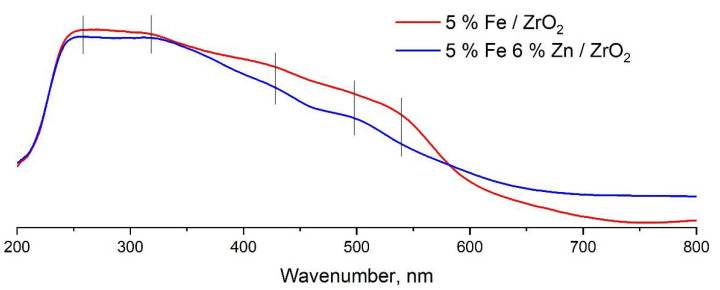
UV/VIS spectra of samples of iron-based catalysts for CO_2_ hydrogenation with and without zinc addition.

UV/VIS spectra of samples of iron-containing catalysts with and without zinc additives are shown in Figure 7. The spectra show the presence of several absorption bands with maxima at 256 nm, corresponding to charge transfer from the nonbonding valence orbital O(2p) to the crystal field orbital Fe(3d) iron atoms in octahedral coordination; at 310 nm, related to the ^6^A_1_→^4^T_1_ dd transition; at 380 nm, corresponding to the ^6^A_1_→^4^E transition; at 410–420 nm, corresponding to the ^6^A_1_→^4^T_2_ transition; and at 520 nm, corresponding to the double transition 2(^6^A_1_)→2(^4^T_1_) [44]. The position of the observed lines in the spectra corresponds to the structure of iron oxide—hematite. The presence of absorption bands in the spectrum at wavelengths less than 350 nm refers to the presence of iron ions Fe^3+^, stabilized in octahedral coordination, and structural iron ions Fe^3+^. The presence of bands at wavelengths above 350 nm can be associated with the presence of rather large massive nanoparticles of iron oxides. In the spectra of the 5%Fe/ZrO_2_ sample, a high intensity of absorption lines above 350 nm is observed, which indicates the presence of rather large particles on the surface of this sample [44]. A decrease in the intensity of these lines in the spectrum of the 5%Fe6%Zn/ZrO_2_ sample may indicate a decrease in the size of iron crystallites due to the stabilizing effect of zinc additives by preventing sintering of small iron particles.

## 3. Discussion

The promotion of the iron-based catalyst with zinc leads to an increase in the activity of the catalyst and the formation of more light C_1_–C_10_ hydrocarbons in the products of the CO_2_ hydrogenation process. At the same time, no significant changes in the CO_2_ conversion was observed. As a rule, the formation of hydrocarbons on iron-containing catalysts proceeds through the stage of CO formation. However, zinc is one of the main components of Cu-containing catalysts for the hydrogenation of CO_2_ into methanol. It can be assumed that, when zinc is introduced into the composition of iron-containing catalysts, it can change the mechanism of CO_2_ hydrogenation towards the formation of hydrocarbons through the formation of methanol. However, the absence of even trace amounts of methanol in the composition of the reaction products contradicts this assumption. The data obtained on DRIFT-CO indicate the appearance of mobile oxygen on the surface of the 5%Fe6%Zn/ZrO_2_ catalyst, which can be attributed to either zinc oxide or ZnFe_2_O_4_. According to the TPR-H_2_ results, it was shown that the introduction of zinc into the composition of the catalyst changes the mechanism of iron reduction, presumably directing it through the stage of FeO formation, which promotes the formation of metallic iron. Taking into account the method of catalyst synthesis and the presence of ZnFe_2_O_4_ lines in the Raman spectrum of the 5%Fe6%Zn/ZrO_2_ sample, it can be assumed that, during the catalyst synthesis, mixed zinc and iron oxide ZnFe_2_O_4_ is formed, the reduction of which leads to the formation of metallic iron through the formation of wustite phase FeO. This leads to the formation of metallic iron Fe0, detected by XPS results, found in the sample after activation and hydrogenation of CO_2_. The presence of named metallic iron is an obligatory intermediate stage in the formation of Hegg carbides, which are the centers of growth and formation of a carbon chain in the processes of hydrogenation of carbon oxides [19]. This explains the increase in the selectivity of the formation of hydrocarbons in the process of hydrogenation of CO_2_ on a catalyst with the addition of zinc. Thus, this indicates that zinc in the iron-containing catalyst is not only a structural, but also an electronic promoter.

The results of UV/Vis spectroscopy suggest the formation of smaller iron particles in the 5%Fe6%Zn/ZrO_2_ catalyst structure, which can be more active in CO_2_ hydrogenation and should be reduced at a lower temperature. However, according to the results of studying the catalyst samples by the TPR-H_2_ method, it can be seen that the reduction of iron is significantly shifted to the high-temperature region. During the synthesis of catalysts, iron and zinc were deposited simultaneously; it can be assumed that, in the case of an unpromoted 5%Fe/ZrO_2_ catalyst, the particles of the supported iron-containing phase can be significantly smaller than the particles of the joint supported phase of iron and zinc in the case of the catalyst 5%Fe6%Zn/ZrO_2_, which makes it difficult for hydrogen to access deep layers of Fe-Zn particles and explains the higher temperature of iron reduction.

## 4. Materials and Methods

### 4.1. Catalyst Preparation

Catalyst samples were prepared by wet-capacity impregnation of the support. Iron (III) nitrate nonahydrate (Fe(NO_3_)_3_×9H_2_O, 99+%, ACROS) was used as a precursor of iron and zinc nitrate monohydrate (Zn(NO_3_)_2_×H_2_O, 99+%, ACROS) was used as a precursor of zinc (ACROS ORGANICS, Geel, Belgium). Distilled water was used as a solvent for the impregnating solution. Zirconium oxide ZrO_2_ promoted with lanthanum oxide was used as a support (Supplementary Materials, Table S1) (Saint-Gobain, Courbevoie, France). The study of the effect of the method of introducing zinc into the structure of an iron-containing catalyst on the catalytic activity in the reaction of CO_2_ hydrogenation was carried out on catalysts of the composition 5%Fe5%Zn/ZrO_2_. These samples were synthesized by the method of co-impregnation of the carrier by incipient wetness and by the method of successive impregnation of the carrier by incipient wetness with aqueous solutions of iron and zinc. The synthesis method by successive impregnation of the carrier with individual solutions of iron and zinc included an intermediate stage of calcination in air at a temperature of 500 °C for 4 h. Monometallic catalysts with compositions of 5%Zn/ZrO_2_ and 5%Fe/ZrO_2_ were also obtained by impregnating the support with an aqueous solution of metal precursors and used as reference samples. To study the effect of the zinc content in the iron-containing catalyst on its properties in CO_2_ hydrogenation by the method of joint incipient wetness impregnation, a series of catalysts with a composition of 5%FeX_Zn_%Zn/ZrO_2_, with a zinc content of 0 to 9%, was synthesized. All catalyst samples were calcined in air at a temperature of 500 °C for 4 h.

### 4.2. CO_2_ Hydrogenation

CO_2_ hydrogenation was carried out in a flow-through catalytic unit equipped with a straight stainless steel reactor with a fixed catalyst bed. During loading, the catalyst sample was mixed with quartz. The samples were activated in a hydrogen flow of 30 mL/min at 500 °C and atmospheric pressure, heating rate 10 °C/min, for 8 h and cooled to room temperature. Then, the flows of hydrogen and carbon dioxide were established, the pressure was raised to the required value, and then the heating was switched on. The ratio of hydrogen and carbon dioxide in the mixture was H_2_/CO_2_ = 2:1. The products were analyzed on a KRISTALL 5000 gas chromatograph (CHROMATEK, Yoshkar-Ola, Russia) equipped with three heat capacity detectors, one flame ionization detector, three M NaX 80/100 packed columns, 2 m × 2 mm, HayeSep R 80/100, 1 m × 2 mm, HayeSep Q 80/100 mesh 1 m × 2 m, and MXT^®^-Alumina BOND/MAPD 30 m × 0.53 mm capillary column (CHROMATEK, Yoshkar-Ola, Russia).

### 4.3. Raman Spectroscopy

To record the Raman spectra of catalyst samples before and after the hydrogenation of CO_2_, a multifunctional automated NT-MDT INTEGRA Spectra system was used (NT-MDT SPECTRUM INSTRUMENTS, Moscow, Russia), equipped with a Cobolt Blues 50 W laser as a radiation source and a 100 × 0.9FN22 objective. A sample weighing about 100 mg was pressed into a tablet without the use of a binder and placed on the instrument stage under a microscope, and the laser was switched on and focused on the sample. The spectra were recorded in the range 200–3000 cm^−1^. The spectra were decoded using the RRUFF database.

### 4.4. Diffuse Reflectance IR Spectroscopy

Diffuse reflectance IR spectra (DRIFT) were recorded at room temperature using a NICOLET protégé 460 spectrometer (Thermo Scientific, Waltham, MA, USA) with a diffuse-reflectance attachment in the range of 6000–400 cm^–1^ with a step of 4 cm^–1^. For a satisfactory signal-to-noise ratio, 500 spectra were accumulated. CaF_2_ powder was used as a standard. Before measuring the spectra, samples in granular form (fraction 50–200 mesh) were subjected to thermal vacuum treatment at a temperature of 450 °C for 2 h (heating rate 5 °C/min) to remove physically adsorbed gases and water. Carbon monoxide was used as a test molecule for the electronic state of metals. Adsorption was carried out at room temperature and equilibrium CO pressure of 15 Torr. The intensity of the bands in the spectra was expressed in Kubelka–Munk units. Data collection and processing were carried out using the OMNIC program (Thermo Scientific, Waltham, MA, USA). The spectra of adsorbed CO were presented as the difference between those recorded after and before adsorption.

### 4.5. Thermoprogrammed Reduction with Hydrogen

TPR-H_2_ measurements were carried out on a semi-automatic setup using a thermal conductivity detector. Here, 100–150 mg of the sample was placed in a quartz U-shaped reactor, in the center of which, in the sample zone, there was a chromel-alumel thermocouple (N.D. Zelinsky Institute of Organic Chemistry RAS, Moscow, Russia ). The sample was preliminarily blown off with Ar (30 mL/min), heating from room temperature to 300 °C at a rate of 10 °C/min and holding at this temperature for 30 min, and cooled in an argon flow to room temperature. Then, a 5% H_2_/Ar mixture (30 mL/min) was fed to the sample and a stable baseline was maintained. After that, the sample was heated using a programmer at a rate of 10 °C/min to 850 °C. To remove the water formed as a result of reduction from the gas phase, a trap was placed between the reactor and the detector, and cooled to –100 °C with a mixture of liquid nitrogen and ethanol. The katharometer signal and temperature were recorded on a computer using an analog-to-digital converter and the Ekochrome software package. The detector was calibrated using CuO reduction (Aldrich-Chemie GmbH, 99%, St. Louis, MO, USA ). All results are normalized to 1 g of sample.

### 4.6. X-ray Photoelectron Spectroscopy

X-ray photoelectron spectra were recorded on a PHI5000VersaProbeII spectrometer (ULVAC-PHI Inc., Kanagawa, Japan). The E_b_ bond energy scale was calibrated using Au4f (83.96 eV) and Cu2p3 (932.62 eV). The E_b_ scale was corrected according to the E_b_ of the Zr3d5 peak (182.2 eV). We used monochromatic Al-Kα radiation (hν = 1486.6 eV) with a power of 50 W. Powder samples of catalysts were fixed on a holder using a special double-sided adhesive tape and placed in a pretreatment chamber. Then, the chamber was evacuated and the rod with the holder was moved into the working chamber of the spectrometer. The residual gas pressure in the working chamber of the spectrometer during the measurement of the spectra was about 10^−9^ Torr. To prevent charging of the sample surface, neutralization was performed with heavy ions of an inert gas. The diameter of the analysis area was 200 µm. Atomic concentrations were determined from survey spectra by the method of relative elemental sensitivity factors. The integral intensities of the following lines were used: C1s, O1s, Zr3d, La3d, and Fe2p3. High-resolution Zr3d spectra were recorded at an analyzer transmission energy of 23.5 eV with a 0.2 eV step, and high-resolution Fe2p spectra were recorded at an analyzer transmission energy of 46.95 eV with a 0.2 eV step. Data collection was carried out using the SpecsLab2 program, and the experimental data were processed using the CasaXPS program (Casa Software Ltd., Teignmouth, UK). Multiplet structures were used to analyze the Fe2p spectrum. In this case, the distance between the peaks, the ratio of intensities, and the difference in FWHM were recorded. Background subtraction was performed using the Shirley method.

### 4.7. Diffuse Reflectance UV/VIS Spectroscopy

Diffuse reflectance UV/VIS spectra were recorded on a Shimadzu UV-3600 Plus spectrophotometer equipped with an ISR-603 integrating sphere (Shimadzu, Kyoto, Japan). The spectra were recorded in the wavelength range of 200–800 nm at room temperature, using BaSO_4_ as a standard and a diluent. The obtained spectra were processed using the UVProbe software (Shimadzu, Kyoto, Japan).

## 5. Conclusions

The effect of zinc addition on the properties of an iron-containing catalyst for the hydrogenation of CO_2_ into hydrocarbons was studied. It was shown that an increase in catalytic activity can only be achieved by supporting iron and zinc from the same solution at the same time. The dependence of the catalyst activity on the Fe/Zn ratio has an extreme character. Samples with an approximately equal ratio of iron and zinc show the highest activity. The presence of zinc in the composition of the catalyst changes the iron reduction scheme and facilitates the easier formation of metallic iron, which is necessary for the formation of active centers responsible for the growth of the hydrocarbon chain. In addition, the presence of zinc in the composition of the catalyst leads to a change in the electronic properties of iron. This indicates that zinc is not only a structural, but also an electronic promoter.

## Figures and Tables

**Table 1:** Hydrogenation of CO_2_ over Fe and/or Zn containing catalysts (H_2_/CO_2_ = 2:1, T = 280 °C, P = 1 atm, 40,000 h^−1^).

Sample	Selectivity, %		Reaction Rate, mol CO_2_×kg_kat_^−1^×h^−1^
	CO	HC	
5%Fe/ZrO_2_ (La)	95	5	8.0
5%Zn/ZrO_2_ (La)	98	2	0.2
Co-impregnation	91	9	16.6
Imp. Fe → 500 °C 4 h on air → Imp. Zn	96	4	3.2
Imp. Zn → 500 °C 4 h on air → Imp. Fe	93	7	5.7

**Table 2:** Influence of zinc content in 5%FeX_Zn_%Zn/ZrO_2_ catalysts on their properties in CO_2_ hydrogenation (H_2_/CO_2_ = 2:1, T = 280 °C, P = 1 atm, 40,000 h^−1^).

Zn Content (X_Zn_), Mass. %	Selectivity, %		Reaction Rate, mol CO_2_×kg_kat_^−1^×h^−1^
	CO	HC	
0	95	5	8.0
1	99	1	9.2
3	97	3	12.6
5	91	9	16.6
7	91	9	16.0
9	92	8	12.0

**Table 3:** The amount of hydrogen absorbed by the samples of iron-containing catalysts with and without zinc addition in the TPR-H_2_ process.

Sample	Specific Absorption of Hydrogen, mol H_2_/g
Carrier ZrO_2_	2.12 × 10^−4^
5%Fe/ZrO_2_	1.28 × 10^−3^
5%Fe6%Zn/ZrO_2_	1.31 × 10^−3^

**Table 4:** Results of XPS study of element content on the surface of 5%Fe/ZrO_2_ (La) and 5%Fe6%Zn/ZrO_2_ (La) catalysts.

Sample	Element Content on the Sample Surface,% wt.					
	C	O	Zr	Fe	La	Zn
5%Fe/ZrO_2_ (La)	12.3	60.8	22.3	2.1	2.6	-
5%Fe6%Zn/ZrO_2_ (La)	13.3	60.8	18.3	1.8	2.4	3.4

**Table 5:** Iron content in different oxidation states on 5%Fe/ZrO_2_ (La) and 5%Fe6%Zn/ZrO_2_ (La) catalysts after catalytic studies.

Sample	Element Content on the Sample Surface,% wt.		
	Fe^0^	Fe^2+^	Fe^3+^
5%Fe/ZrO_2_(La)	-	48	52
5%Fe6%Zn/ZrO_2_ (La)	3	47	50

## Data Availability

Data available in a publicly accessible repository that does not issue DOIs. Publicly available datasets were analyzed in this study. This data can be found here: [https://rruff.info/] (accessed on 28 January 2022).

## Supplementary Materials

The following are available online, Figure S1: XPS spectra of catalyst samples 5%Fe/ZrO_2_ (**A**) and 5%Fe6%Zn/ZrO_2_ (**B**); Figure S2. Raman spectra for the spent catalysts after the CO_2_ hydrogenation; Table S1: Characteristics of the used catalyst carrier ZrO_2_.

## Sample Availability

Samples of the catalysts are available from the authors.
