# Supplementary material for: Zn Doping Effect on the Performance of Fe-Based Catalysts for the Hydrogenation of CO2 to Light Hydrocarbons
Source: Molecules. 2022 Feb 4;27(3):1065. doi: 10.3390/molecules27031065 (PMC8838917; doi:10.3390/molecules27031065)
Supplement: Supplementary file 1 [file molecules-27-01065-s001.zip › molecules-1561081-supplementary.pdf]

**Supplementary Materials:**

**Table S1.** Characteristics of the used catalyst carrier $ZrO_2$.

| | |
|---|---|
| Manufacturer | Saint-Gobain |
| Compound | 70–100 % $ZrO_2$ + 5–15% $La_2O_3$ |
| Surface area $S_{BET}$, м$^2$/г | 108 |
| Pore volume $V_{total}$, cm$^3$/g | 0.234 |
| Mesopore volume $V_{meso}$, cm$^3$/g | 0.234 |
| Granule size, mm | 0.25–0.50 |
| Bulk density, g/cm$^3$ | 1.3 |

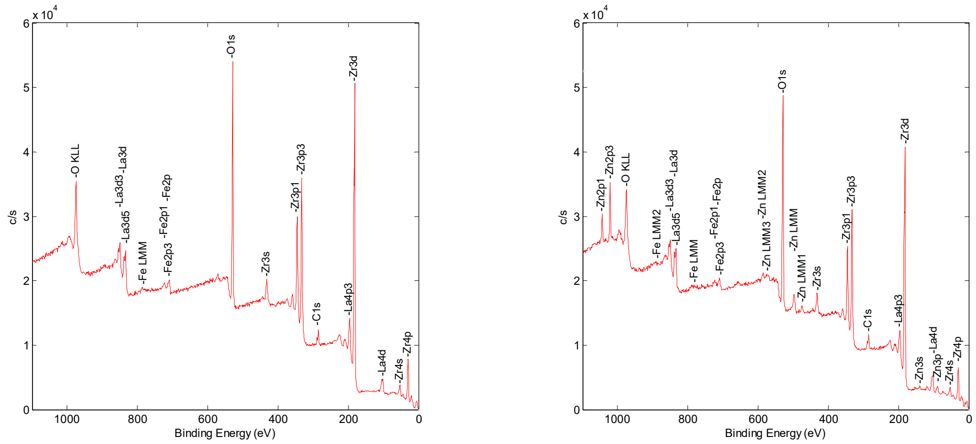


(**A**) (**B**)

**Figure S1.** XPS spectra of catalyst samples 5%Fe/$ZrO_2$ (**A**) and 5%Fe6%Zn/$ZrO_2$ (**B**).

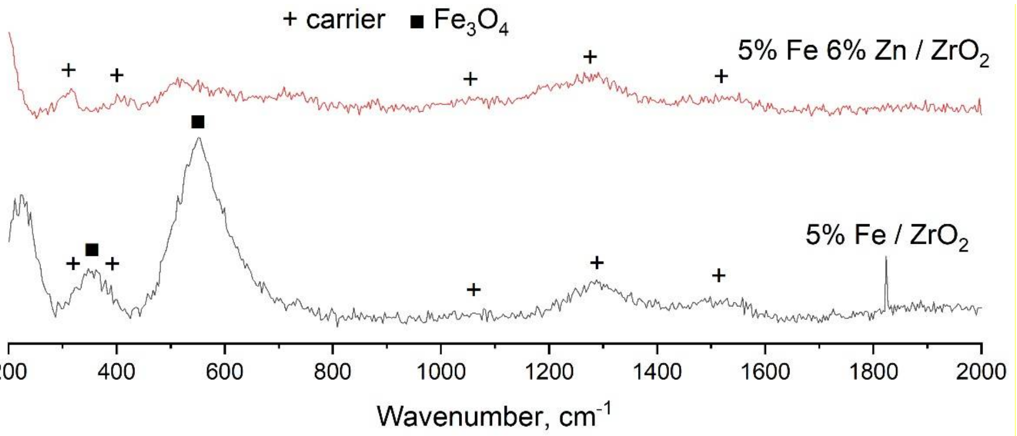


**Figure S2.** Raman spectra for the spent catalysts after the $CO_2$ hydrogenation.
